# Hepatitis B immunisation and immune status of nurses in a regional hospital in central South Africa

**DOI:** 10.4102/safp.v66i1.5871

**Published:** 2024-06-26

**Authors:** Emily M. Makola, Willem H. Kruger, Perpetual Chikobvu

**Affiliations:** 1 Department of Community Health, Faculty of Health Sciences, School of Clinical Medicine, University of the Free State, Bloemfontein, South Africa

**Keywords:** hepatitis B, vaccine, nurses, immune status, South Africa

## Abstract

**Background:**

The hepatitis B virus (HBV) is one of the most important biological occupational hazards for healthcare workers. A high percentage of HBV infections are attributable to percutaneous occupational exposure. This study aimed to describe the HBV immunisation and current immune status of all the nurses employed in a regional hospital in central South Africa.

**Methods:**

A descriptive record review included all the nurses (*N* = 388) employed in a regional hospital in central South Africa from 01 January 2018 to 31 January 2020. A total of 289 health records were included in the study. Data were analysed using descriptive statistics. Logistic regression analysis was used to establish factors associated with full immunisation.

**Results:**

Most nurses were females (87.9%), working in medical (27.0%) wards. Only 20.4% of nurses received one dose of vaccine, while 51.2% received the three prescribed doses. However, 91.2% of nurses did not receive the vaccine at the correct intervals. Most of the tested nurses (71.0%) were immune. Immunisation status was significantly associated with religion (*p* < 0.001) and schedule (*p* = 0.003). Nurses who were non-Christians were 35.9% less likely to be fully vaccinated compared to Christians.

**Conclusion:**

Half of the nursing staff received three doses as prescribed. All nurses should receive the vaccine against HBV and their immune status monitored to minimise the risk of an infection. It is therefore recommended that proof of immunity should be a requirement.

**Contribution:**

This study found a high percentage of nurses with HBV antibodies, which will ensure workplace safety.

## Introduction

Viral hepatitis is the seventh leading cause of death worldwide, with hepatitis B and C being the most serious, causing around 96% of hepatitis-related deaths.^1^ Different degrees of endemicity are recognised worldwide, and the transmission of the virus is evident in countries where the disease is highly endemic and within households containing HB surface antigen (HBsAg) carriers.^2^ Infection with the hepatitis B virus (HBV) may result in acute or chronic disease, both of which can be asymptomatic, and the severity of the disease ranges from unapparent cases (detectable only by liver function tests) to fulminant, fatal disease in the form of liver cirrhosis and failure or hepatocellular carcinoma.^3^ A major public health concern is that 5% – 10% of infected people cannot clear the virus and become chronically infected.^4^ In addition, chronic hepatitis B is divided into five stages with no formal agreement on the fifth stage (the four stages being immune tolerance [IT], immune clearance [IC], immune control [ICO], and immune reactivity [IR]), with the first stage that can last for weeks or decades. This first stage is characterised by a very high load of viruses and antigenemia in the absence of any disease. At the same time, the occult hepatitis B infection represents a phenomenon of genetic heterogeneity in genomic regulatory elements that results in undetectable HBsAg with either positive or negative anti-hepatitis B core (anti-HBc) antibodies in serum.^5,6^

Hepatitis B is a biological hazard for the general population, and it is one of the main biological occupational hazards, putting healthcare workers at risk of contracting the infection as they are frequently handling infected blood or body fluids.^7,8^ Hepatitis B virus transmission may occur via several routes. The most frequent method of exposure in healthcare workers in the healthcare setting is injuries with sharp objects such as needles contaminated with blood.^7,9^ It is stated that 40% – 65% of HBV infections among healthcare workers in developing countries are attributable to percutaneous occupational exposure compared to the corresponding risk of 10% in developed countries.^10^ Hepatitis B virus is one of the most efficiently transmissible viruses following a percutaneous exposure with transmission via a needlestick injury of approximately 2% with HBVe antigen (HBeAg)-negative blood and 19% with HBeAg-positive blood.^11^

While HBV is preventable through vaccination, available treatment for HBV infection does not provide a complete cure. Therefore, prevention is a crucial approach when dealing with this biological hazard. All healthcare workers should be ideally vaccinated as a precaution before entering the healthcare workforce.^7,12^ As the HBV vaccine was developed in 1982, it is considered the cornerstone of HBV infection control, and it is safe, effective and highly acceptable.^12,13^ Primary vaccination in immunocompetent adults consists of a three-dose series.^14^ The mentioned three-dose series is administered at 0 months, 1 month and 6 months and offers a 95% – 99% protective immune response with proven long-term efficacy.^7^ Four doses may be given for programmatic reasons, administered according to the schedules of national routine immunisation programmes.^15^ Despite scientific evidence of protective immune response, the vaccine uptake among healthcare workers in the developing world is low.^12^ A meta-analysis study concluded that only a quarter of African healthcare workers are fully vaccinated.^14^ The percentage of healthcare workers fully vaccinated in South Africa is no better. A study confirmed that 12.0% – 40.0% of the 333 healthcare workers in two South African provinces were fully vaccinated.^16^ Results also showed that over 70% received at least one dose of the HBV vaccine.^16^ Recall bias should be considered while examining such results, as healthcare workers cannot remember whether they were vaccinated.^17^

Literature shows that occupational exposure to blood and body fluids and percutaneous injury among African nurses consistently occurred.^8,12,14^ This has profound implications because of the possibility of occupational exposure to blood-borne HBV. To mitigate this, the *South Africa Occupational Health and Safety Act* of 1993 stipulates that in any incident where a healthcare worker is exposed to the virus, such incident must immediately be reported to the employer and the health and safety representative at the workplace.^18^ In addition, the national guideline for the management of viral hepatitis guides further management of the case.^19^

Vaccination of nurses and recording of vaccination status and immune response are critical to ensure that nurses receive optimal care.^2^ In South Africa, the *Occupational Health and Safety Act* of 1993 indicates the need for the employer to keep all relevant records of risk assessment, medical surveillance and exposure monitoring.^18^ However, there is still a need to monitor immunity against HBV, especially for healthcare workers. Therefore, there is a need to research the HBV immunity status among the nursing category in the regional hospitals in South Africa. These results can have practical implications for the health status of exposed nurses because of the high probability of HBV transmission, putting more pressure on the availability of nurses as a scarce human resource for healthcare in Africa.^20^

This research aimed to describe the HBV immunisation and current immune status of all nurses employed in a regional hospital in Central South Africa

## Methods

### Study design and setting

A descriptive record review study was conducted using personal health records of nurses of all categories employed between 01 January 2018 and 31 January 2020 at the Bongani Regional Hospital, Welkom. This regional hospital has 420 beds and serves five district hospitals within the Lejweleputswa District municipality of the Free State, South Africa.

### Data collection

A health record file is maintained and stored for each nurse by the occupational health personnel at the hospital’s occupational health clinic. Sociodemographic information included age, gender, marital status, category of nursing, work experience and all relevant health information, including vaccination history and immunisation status, which are recorded routinely in these records. A pilot study was conducted on five nurses’ files to determine the feasibility of the study. No changes were made as a result. The files that met the inclusion criteria were included in the study consecutively as they were retrieved. All the information was collected through record review. A record was considered for review if age, gender and nursing category were recorded. Records with these three variables missing were excluded from the study. This demographic information was vital as it would assist the institution with possible intervention strategies.

### Data analysis

Independent sociodemographic variables considered for analysis included gender (male or female), age (categorised as 21–29 years, 30–50 years or > 50 years), category of nurses (professional nurses, assistant nurses or enrolled nurses), work experience (categorised as 0–10 years, 10–20 years or > 20 years), marital status (married or unmarried), religion (Christian or other, i.e. non-Christian) and workstation (causality, maternity, surgery, medical, paediatric or theatre). Independent immunisation characteristics considered were dose (1st dose, 2nd dose, or 3rd dose), booster (yes or no), schedule (yes or no) and antibody titre > 10 IU/L (yes or no). The outcome variable used was defined as immunisation status (received three HBV doses [fully immunised] or received < 3 HBV doses [not fully immunised]).

Data were analysed using Stata version 12, using descriptive statistics such as frequencies and percentages as well as graphical presentation. All the nurses’ files included in the study indicated that the nurses had received at least the first dose of the HBV vaccine. Gender, age and category of nursing differences in immunisation characteristics were compared using a Chi-square test. Pearson’s Chi-square (χ^2^) test was used to establish any association between independent variables and the outcome variable. The data were also subjected to univariate and multivariate logistic regression analysis to establish factors associated with full immunisation. The odds ratios (ORs) and their corresponding 95% confidence intervals (CIs) were estimated and the significance level considered for the analysis was 0.05 or less.

The backward stepwise elimination method was used in the multivariate logistic regression analysis. Initially, all covariates (sociodemographic and immunisation characteristics) were included in the model. At each stage, the significance of the inclusion of the covariates in the model was examined using the Wald test. Variables that were not contributing significantly (*p* > 0.05) to the model were then removed depending on the magnitude of the *p*-value. However, the results of the full model are presented, although only two variables had a *p* < 0.05.

All the nurses’ files included in the study indicated that the nurses had received at least the first dose of the HBV vaccine, and the characteristics of the nurses and completeness of HBV immunisation were described using descriptive statistics.

### Ethical considerations

Ethical approval to conduct this study was obtained from the Health Sciences Research Ethics Committee, Faculty of Health Sciences of the University of the Free State (reference no.: UFS-HSD2019/2015/2801). The Free State Department of Health and the Management of the Bongani Regional Hospital gave permission to access the personnel files. As the Provincial Department of Health owns the personnel files, only departmental approval was required and informed consent from the nurses was not needed.

The first author worked in the occupational health clinic during the study. Data were collected in 2020 from the personnel health files and recorded on an Excel spreadsheet by the primary researcher. Confidentiality of the information gathered from the clinical records was assured using a research numbering system. Identifiable information such as names and surnames or ID/Persal (i.e., personnel number on the electronic data system for public servants) was not recorded. Only the researcher had access to the number system on a password-protected computer.

## Results

The health records of 388 nurses were reviewed. A total of 289 files met the inclusion criteria and were included in the study.

All the 289 records of nurses reviewed indicate that the nurse received at least one dose of the HBV vaccine. For this study, fully vaccinated/fully immunised was defined as having received all three HBV vaccine doses. Otherwise, the nurse is not considered fully vaccinated/fully immunised. Of the 289 records included in the analysis, 51.2% indicated that they had received three doses of HBV, and 48.9% had received < 3 doses.

Of the 289 nurses, 87.9% were females and 56.7% were between 30 and 50 years (Table 1). Three-quarters (76.8%) of the files were for either professional or enrolled nurses. Most (61.3%) nurses had over 10 years of work experience, whereas 56.1% were married and 66.1% were Christians.

**TABLE 1:** Distribution of sociodemographic, immunisation characteristics by hepatitis B virus immunisation status (*N* = 289).

Variable name	Total		Immunisation status				χ^2^ *p*-value
	*n*	%	Not fully immunised (*n* = 141)		Fully immunised (*n* = 148)		
			*n*	%	*n*	%	
**Gender**							0.739
Male	35	12.1	18	12.8	17	11.5	
Female	254	87.9	123	87.2	131	88.5	
**Age (years)**							0.377
21–29	19	6.6	10	7.1	9	6.1	
30–50	164	56.7	85	60.3	79	53.4	
> 50	106	36.7	46	32.6	60	40.5	
**Category of nurses**							0.647
Assistant nurses	67	23.2	36	25.5	31	20.9	
Professional nurses	111	38.4	53	37.6	58	39.2	
Enrolled nurses	111	38.4	52	36.9	59	39.9	
**Work experience (years)**							0.623
0–10	112	38.8	57	40.4	55	37.2	
10–20	102	35.3	51	36.2	51	34.5	
> 20	75	26.0	33	23.4	42	28.4	
**Marital status**							0.059
Married	127	43.9	54	38.3	73	49.3	
Unmarried	162	56.1	87	61.7	75	50.7	
**Religion**							< 0.001
Christian	191	66.1	77	54.6	114	77.0	
Other	98	33.9	64	45.4	34	23.0	
**Immunisation characteristics**							
Dose (*n* = 289)							< 0.001
1st dose	59	20.4	59	41.8	0	0.0	
2nd dose	82	28.4	82	58.2	0	0.0	
3rd dose	148	51.2	0	0.0	148	100.0	
Booster (*n* = 27)							-
Yes	12	44.4	0	0.0	12	44.4	
No	15	55.6	0	0.0	15	55.6	
Schedule (*n* = 228)							0.003
Yes	20	8.8	1	1.3	19	12.8	
No	208	91.2	79	98.7	129	87.2	
Antibody titre > 10 IU/L (*n* = 214)							0.002
Yes	152	71.0	62	60.8	90	80.4	
No	62	29.0	40	39.2	72	19.6	

A total of 148 nurses were fully vaccinated. None of the sociodemographic variables was statistically significantly associated with HBV vaccination status (*p* > 0.05), except for religion (*p* < 0.05), with Christians being more likely to be fully vaccinated (77.0%). However, marital status was close to statistically significant (*p* = 0.059) (Table 1). Only 8.8% of the nurses received the vaccine on schedule and 29% did not have antibody titre > 10 IU/L.

**FIGURE 1:**
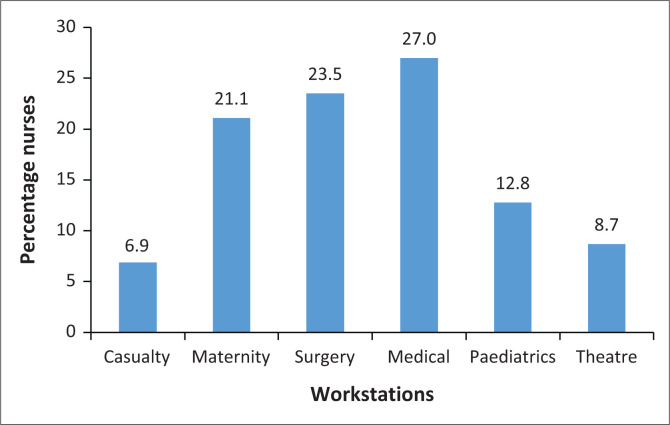
Distribution of nurses at different workstations (*N* = 289).

The highest percentage of nurses worked in the medical (27.0%), surgical (23.5%) and maternity (21.1%) wards, with the casualty ward being represented by 6.9% (Figure 1).

**FIGURE 2:**
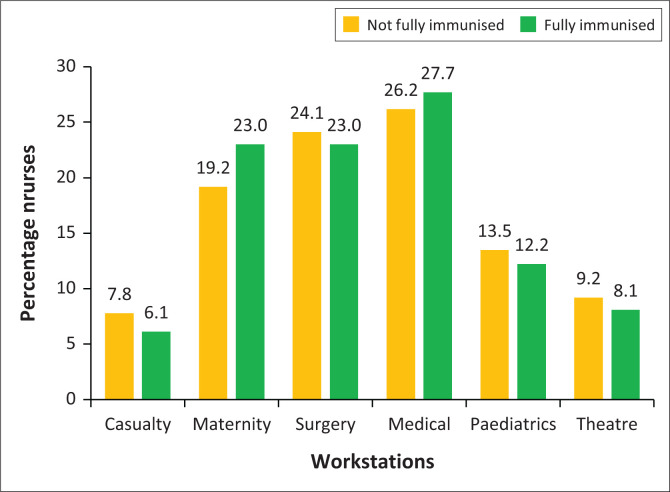
Comparison of immunisations across workstations (*N* = 289).

Figure 2 shows the comparison of HBV vaccination status across the workstations. Slightly more nurses were fully vaccinated in maternity (23.0% vs. 19.2%) and medical (27.7% vs. 26.2%) compared to those not fully vaccinated. Workstation was not statistically significantly associated (*p* = 0.954) with vaccination status.

Nearly half (51.2%) received the three doses as prescribed, with 20.4% receiving only one dose of vaccine (Table 2). Of the 289 nurses, only 12 (4.2%) received the booster dose. No HBV vaccine side effects were recorded. Seventy-four per cent (*n* = 214) of nurses had their hepatitis B antibody titre checked, of which 71.0% proved to have a titre > 10 IU/L. A higher percentage of female nurses received the third dose than male nurses (51.6% vs. 48.6%). Only one male (3.5%) compared to 19 females (9.6%) received the vaccines on schedule. More female nurses also had a higher percentage of antibody titre > 10 IU/L than male nurses (72.3% vs. 60.9%). None of the differences between male and female nurses were statistically significant (*p* > 0.05) (Table 2).

**TABLE 2:** Comparison of the hepatitis B virus immunisation characteristics by gender.

Immunisation characteristics	Total		Male (*n* = 35)		Female (*n* = 254)		χ^2^ *p*-value
	*n*	%	*n*	%	*n*	%	
**Dose (*n* = 289)**							0.686
1st dose	59	20.4	6	17.1	53	20.9	
2nd dose	82	28.4	12	34.3	70	27.6	
3rd dose	148	51.2	17	48.6	131	51.6	
**Booster (*n* = 27)**							0.411
Yes	12	44.4	2	66.7	10	41.7	
No	15	55.6	1	33.3	14	58.3	
**Schedule (*n* = 228)**							0.278
Yes	20	71.0	1	3.5	19	9.6	
No	208	29.0	28	96.6	180	90.5	
**Antibody titre > 10 IU/L (*n* = 214)**							0.256
Yes	152	71.0	14	60.9	138	72.3	
No	62	29.0	9	39.1	53	27.8	

Of the 111 professional nurses, 53.2% received the three doses as prescribed, compared to 46.3% for assistant nurses and 53.2% for enrolled nurses. Only nine professional nurses received a booster dose, whereas 11 enrolled nurses also received a booster dose. More than 80% of nurses were not on schedule for all three categories of nursing. The majority of nurses in each of the categories had antibody titre > 10 IU/L, 72.3%, 73.8% and 67.9% for professional nurses, assistant nurses and enrolled nurses, respectively. Dose (*p* = 0.774) and antibody titre > 10 IU/L (*p* = 0.700) were not statistically significantly associated with the category of nursing, whereas booster (*p* = 0.020) and schedule (*p* = 0.006) were significantly associated with the category of nursing. Approximately 57% of nurses over 50 years received three doses, compared to 48.2% for those nurses in the 30–50 years age group. Only one nurse in the 21–29 years age group and 16 nurses in the 30–50 years age group received a booster. More than 90% of the nurses in each age group were not on schedule with their HBV immunisation. Nearly 71% of nurses in the 30–50 years age group and above 50 years had antibody titre > 10 IU/L. None of the immunisation characteristics were significantly (all *p*-values > 0.05) associated with age groups (Table 3).

**TABLE 3:** Comparison of hepatitis B virus immunisation characteristics by the nursing categories and age groups.

Immunisation characteristics	Category of nursing						χ^2^ *p*-value	Age (years)						χ^2^ *p*-value
	Professional nurse		Assistant nurse		Enrolled nurses			21–29		30–50		> 50		
	*n*	%	*n*	%	*n*	%		*n*	%	*n*	%	*n*	%	
**Dose (*n* = 289)**	111	-	67	-	111	-	0.774	19	-	164	-	106	-	0.659
1st dose	24	21.6	13	19.4	22	19.8	-	5	26.3	34	20.7	20	18.9	-
2nd dose	28	25.2	23	34.3	31	27.9	-	5	26.3	51	31.2	26	24.5	-
3rd dose	59	53.2	31	46.3	58	52.3	-	9	47.4	79	48.2	60	56.6	-
**Booster (*n* = 27)**	9	-	7	-	11	-	0.020	1	-	16	-	10	-	0.629
Yes	6	66.7	0	0.0	6	54.5	-	0	0.0	7	43.8	5	50.0	-
No	3	33.3	7	100.0	5	45.5	-	1	100.0	9	56.3	5	50.0	-
**Schedule (*n* = 228)**	87	-	52	-	89	-	0.006	14	-	128	-	86	-	0.488
Yes	3	3.5	10	19.2	7	7.9	-	0	0.0	12	9.4	8	9.3	-
No	84	96.5	42	80.8	82	92.1	-	14	100.0	116	90.6	78	90.7	-
**Antibody titre > 10 IU/L (*n* = 214)**	65	-	65	-	84	-	0.700	19	-	123	-	72	-	0.961
Yes	47	72.3	48	73.8	57	67.9	-	13	68.4	88	71.5	51	70.8	-
No	18	27.7	17	26.2	27	32.1	-	6	3.6	35	28.5	21	29.2	-

The religion, schedule and antibody each had a statistically significant influence on fully vaccinated (*p* < 0.05) (Table 4). Nurses who were non-Christians were 35.9% less likely to be fully vaccinated compared to Christians, and similarly, nurses who had a schedule of between two and three doses were 8.6% less likely to be fully vaccinated compared to those with a schedule between one and two doses and those nurses without antibody titre > 10 IU/L were 37.9% less likely to be fully vaccinated compared to those nurses with antibody titre > 10 IU/L.

**TABLE 4:** Factors associated with hepatitis B virus immunisation (*n* = 289).

Variables	Categories	Univariate			Multivariate		
		Crude OR	*p*	95% CI	Adjusted OR	*p*	95% CI
Gender	Female *(Ref*)	1	-	-	1	-	-
	Male	1.128	0.739	0.556; 2.287	1.655	0.403	0.508; 5.391
Age (years)	21–29 *(Ref)*	1	-	-	1	-	-
	30–50	1.033	0.947	0.399; 2.673	0.477	0.293	0.120; 1.898
	> 50	1.449	0.458	0.545; 3.857	0.500	0.404	0.098; 2.546
Religion	Christian *(Ref)*	1	-	-	1	-	-
	Other	0.359	< 0.001	0.216;0.596	0.349	0.008	0.159; 0.764
Marital status	Married *(Ref)*	1	-	-	1	-	-
	Not married	0.638	0.060	0.399; 1.018	0.604	0.196	0.281; 1.298
Nursing category	Professional nurse *(Ref)*	1	-	-	1	-	-
	Assistant nurse	0.759	0.374	0.413; 1.394	0.611	0.344	0.220; 1.697
	Enrolled nurse	0.965	0.893	0.569; 1.634	0.950	0.916	0.371; 2.433
Work station	Casualty *(Ref)*	1	-	-	1	-	-
	Maternity	1.539	0.405	0.558; 4.249	1.552	0.64	0.246; 9.808
	Surgery	1.222	0.694	0.449; 3.326	2.201	0.391	0.363; 13.361
	Medical	1.354	0.547	0.505; 3.633	1.238	0.809	0.218; 7.021
	Paediatrics	1.158	0.792	0.389; 3.449	0.968	0.973	0.149; 6.277
	Theatre	1.128	0.841	0.347; 3.671	0.946	0.958	0.120; 7.484
Work experience (years)	0–10 *(Ref)*	1	-	-	1	-	-
	10–20	1.258	0.552	0.591; 2.676	0.977	0.959	0.402; 2.375
	> 20	1.785	0.193	0.746; 4.268	1.386	0.634	0.362; 5.310
Schedule	Yes *(Ref)*	1	-	-	1	-	-
	No	0.086	0.018	0.011; 0.655	0.058	0.012	0.006; 0.535
Antibody titre > 10 IU/L	Yes *(Ref)*	1	-	-	1	-	-
	No	0.379	0.002	0.205; 0.699	0.637	0.331	0.256; 1.582

CI, confidence interval; OR, odds ratio; Ref, reference group.

After controlling for other variables, the multivariate analysis still revealed that religion and schedule were the only significant (*p*-values < 0.05) factors influencing full vaccination. Non-Christians were still less likely to be fully vaccinated than Christians (adjusted OR [AOR]: 0.349, 95% CI: 0.159–0.764). Nurses with a schedule of between two and three doses were still less likely to be fully vaccinated than those with a schedule between one and two doses (AOR: 0.058, 95% CI: 0.006–0.535). Nurses without antibody titre > 10 IU/L were no longer significantly different in vaccination status from nurses with antibody titre > 10 IU/L (AOR: 0.331, 95% CI: 0.256–1.582) (Table 4).

## Discussion

This study describes the HBV immunisation and immune status of nurses in a regional hospital in Welkom, Free State. The study found that all the nurses had received at least one dose of HB vaccine. This figure is higher compared to a study conducted in Gauteng province, where 71.0% of healthcare workers received at least one dose of the vaccine.^21^ Hepatitis B virus vaccine coverage is generally low in Africa. A systematic review consisting of 35 studies from 15 African countries found the partial (received one or two doses) vaccination coverage in Africa to be 17.8% and the full HBV coverage at 24.7%, with the highest coverage from North Africa at 62.7% and the lowest coverage of 13.4% from central Africa.^14^

Hepatitis B virus is a biological hazard, especially for healthcare workers at high risk.^12^ With the HBV vaccine as a preventative measure, Puro et al.^22^ highlighted health professionals should be vaccinated against HBV before they start working.^22^ Furthermore, Coppeta et al.^23^ found that many healthcare workers had a non-protective anti-HBs titre at their first employment.^23^ However, it was not possible with this study to confirm that the first vaccination was received before the nurses started working in the hospital. The results of this study showed that 20.4% (*n* = 59/289) of nurses received only the first HBV vaccination dose, and 51.2% of the nurses were fully immunised with 90 (59.2%) out of 152 nurses who had protective ant-HBs titre, had received three doses. These results are higher than reported by Razwiedani et al.,^21^ where 49.0% of healthcare workers were fully vaccinated and only 11.0% reported to be protected against HBV.^21^ Furthermore, our results contradict the suggestion by the CDC advisory committee on immunisation practice that after one dose, an approximate protection rate of 30% – 55% can be expected, while more than 90% protection can be expected after the third dose.^24^ Only 71% of the 214 nurses showed an antibody titre of the accepted 10 IU/L titre. This low percentage of nurses that have protective levels of antibodies against HBV in this study is in contrast with similar studies indicating that between 83%^25^ and 99.6%^26^ of healthcare workers developed protective antibody levels after the primary immunisation schedule.

The CDC recommends a comprehensive vaccination schedule against HBV consisting of three doses at 0 months, 1 month and 6 months.^24^ This study found an increase among nurses who received only the first dose (20.4%) compared to those who received all three vaccine doses (51.2%). This indicates that just over half of the nurses completed the recommended three doses of the HBV vaccine in this study. However, similar results were found in a teaching hospital in Ethiopia, where the increase was from 33.6% (first dose) to 44.5% (all three doses).^27^ This uptake of vaccination is in contrast with the rest of Africa where it was found that the full vaccination coverage of nurses was 26.3% in a systematic review of 15 African countries.^14^ The results confirm the non-adherence to the vaccination schedule as proposed by the CDC.^24^ This study shows that 29.0% of nurses did not receive their vaccine on schedule and were 8.6% less likely to be fully immunised than those who received the vaccine on schedule. Wijayadi et al.^28^ highlighted that healthcare workers have a high exposure rate to HBV, and the effect of non-adherence to the scientifically proven and accepted immunisation schedule can further increase the risk of acquiring HBV,^28^ which is associated with irreversible health-related consequences. It is, therefore, unnecessary to have an increased risk of acquiring HBV as it can be dramatically reduced by having fully vaccinated nurses working in various areas of the hospital, given that the HBV vaccine is provided to healthcare workers free of charge.^19^

This study found that 62.9% of nurses with over 10 years of work experience were fully vaccinated, and nurses who had 10–20 years and those with > 20 years of work experience were 1.26 times and 1.79 times more likely to be fully immunised, respectively. These results are similar to what was found in Gauteng, where nursing staff and those with > 10 years of work experience were 2.5 and 2.6 times more likely to be vaccinated, respectively.^21^ A study conducted in Sierra Leone found that healthcare workers with more work experience had more knowledge about preventive measures than those with less work experience.^29^ In this study, more than one-third (38.8%) of nurses had less than 10 years of working experience, which could also contribute to the low rate of fully vaccinated nurses (51.2%).

This study also found that more men had non-protective antibody titre levels than women (39.1% vs. 27.8%) after immunisation. This result is supported by Trevisan et al.,^30^ who found that gender affects the immune response to the HBV vaccine, with females showing a 1.21-fold increase in median antibody titre.^30^

Our study found that a higher proportion of older nurses were fully vaccinated than younger nurses, 40.5%, 53.4% and 6.1%, for > 50 years, 30–50 years and 21–29 years, respectively. In addition, multivariate analysis results show that nurses > 50 years old were 0.500 times, and 30–50-year-olds were 0.477 times less likely to be fully immunised compared to 21–29-year-olds, although the difference was not significant. These results are similar to what was observed in a study conducted in a Brazilian university hospital where they found that the association between vaccination and positive anti-HBs serology demonstrated that the chance of immunisation among healthcare workers was smaller with increasing age (multivariate analysis, OR: 0.978; 95% CI: 0.961–0.996; *p* = 0.014).^31^

Different opinions still exist on whether antibody levels should be measured after immunisation.^32^ A study conducted in Japan highlighted that booster doses should be administered because of their effectiveness if administered with the same schedule as the primary vaccination.^32^ Despite the effectiveness of the HBV vaccine, one study reported that between 5% and 10% of some healthy individuals do not respond satisfactorily to vaccination against hepatitis B,^33^ and it is therefore important to consider a booster dose. Although 71% of the nurses in our study had protection, only 12 received a booster dose. It was, however, not possible to conclude the impact of the booster dose on the antibody levels of the 12 nurses.

A study^23^ conducted among healthcare workers in Italy found that a protective anti-HBs titre of > 10 mIU/mL was observed in almost 90% of subjects receiving the booster. On the other hand, Cocchio et al.^26^ concluded that, even without a booster dose, there is good persistence of protective anti-HBs titres in healthcare workers up to 30 years after a primary vaccination cycle. These conclusions were confirmed by a study on 300 participants showing that 83% had protective hepatitis B antibody levels (≥ 10 mIU/mL), regardless of whether one gets a booster dose.^25^ The high percentage (29%) of non-protective antibody levels among nurses in this study and the conclusion made by Cocchio et al.^26^ deepen the debate on whether to measure the antibody levels against HBV after a healthcare worker is fully vaccinated and whether all healthcare workers should receive a booster.

Vaccine safety has been occasionally questioned; however, it is stated that HBV vaccines are well tolerated with mild side effects, which are usually confined to the injection site. Nevertheless, healthcare workers are still uncertain of the safety of the HBV vaccine.^34^ Misinformation on social and mainstream platforms can contribute to these concerns. For example, 17 deaths and one case of anaphylactic shock were reported in China on these platforms related to an HBV vaccine.^35^ Despite the small number of nurses in this study, the results confirmed the safety of the HBV vaccine, as no side effects were recorded. It should be noticed that the Department of Health of South Africa provides HBV immunisation at no cost to all healthcare workers.^19^ Furthermore, the HBV vaccine was incorporated into the national Expanded Programme on Immunisation (EPI) at 6 weeks, 10 weeks and 14 weeks of age.^36^

### Recommendations

This study’s low rate of nurses who received all the vaccines as stipulated in the HBV immunisation programme reflects the need for an effective immunisation programme to be implemented at an institutional level that will intensify the increased compliance rate with the recommended three vaccine doses. In addition, the mentioned low rate of nurses who received all the vaccines as stipulated and the high exposure rate to HBV, we recommend that the hospital need to implement the Regulations for Hazardous Biological Agents, in totality, by developing a standard operating procedure aimed at implementing this regulation. The implementation of the mentioned regulation needs to focus on the following:

- Continuous risk assessment of exposure for the nurses.
- Regular exposure monitoring and implementation of medical surveillance.
- Consistent provision of personal protective equipment.
- Continuous training of employees on the risk and implications of HBV infections as well as creating awareness on the need for HBV vaccination.

Compliance with the mentioned regulation can be achieved through the establishment and implementation of formal systems such as mandatory vaccination for all healthcare workers against HBV before they start working should be implemented. Also, the human resource department and the occupational health service of the hospital could play a pivotal role by ensuring that proof of vaccination is submitted before a healthcare worker is allowed to start working at the hospital. Furthermore, supervisors of healthcare workers at the hospital can assist by ensuring their employees adhere to the immunisation schedule. In contrast, the occupational health services in the hospital can do the necessary follow-ups in good time.

More emphasis should be placed on HBV infection prevention using in-house training programmes for younger or less experienced nurses and male nurses to educate them about the risk of acquiring HBV and the need to be fully vaccinated.

### Limitations

Study participants were restricted to nurses only, and they comprise only a portion of healthcare workers, and as a result, membership bias may have been present. Although a record review allows the research to be completed early, the design has some limitations. The completeness of records was also an issue. Missing or undocumented information could have contributed to the study as the researchers reviewed existing records.

The duration of employment in the hospital was not collected in this study, which contributes to the incompleteness of the records as some of the records may not have been updated on the vaccination status of the nurses if they had just joined the hospital. The other limitation is that the accuracy of the information collected during the record review provides only known information based on the clients’ visits to the occupational health clinic. The information may not reflect an individual’s current HBV immunisation status.

Some records (pages of the patient file) may have been incomplete or lost over time, leading to missing data and resulting in the exclusion of the patient file. Another limitation is that patient files are created for various health purposes and by different healthcare workers. Therefore, interpreting the content within these files can be subjective, especially when there is a lack of corroborating evidence. Lastly, the reviewed records were based on patient visits to the clinic for any other purpose and HBV vaccination recording may be prone to recall and social desirability biases.

## Conclusion

Sufficient occupational health and safety measures must be implemented at Bongani Regional Hospital to ensure that all healthcare workers are vaccinated according to the national Regulations for Hazardous Biological Agents.^18^ All nurses must receive the recommended three doses of the HBV vaccine and have their antibodies tested to ensure their safety at work. A booster needs to be encouraged for the nurses who usually work in areas of the hospital where there is a high risk of exposure to HBV. A routine assessment and evaluation of the employee’s medical and occupational history, a physical examination and biological tests, including HBV and hepatitis B antibodies, need to be initiated as required by the regulations.

Continuous training programme on the risks and implications of HBV infection is needed for hospital employees. The hospital needs to start an HBV vaccine compliance monitoring system for both new and existing healthcare workers, including strict compliance for protection in risky occupational procedures. Serological tests to assess anti-HBs titre need to be incorporated into routine practices by occupational health clinics and make the risks of HBV infection known, including the need for preventive measures (universal precaution and vaccination). Targeted education, focusing mainly on male healthcare workers, is needed as this is fundamental to protect healthcare workers against HBV infection. Finally, the recording of information in the patient files by occupational health nurses should be strengthened to ensure accurate and complete information in the patient file.
